# Structure-guided sequence representation learning for generalizable protein function prediction

**DOI:** 10.1093/bioinformatics/btaf511

**Published:** 2025-09-14

**Authors:** SeokJun On, Yujin Jeong, Eun-Sol Kim

**Affiliations:** Department of Artificial Intelligence, Hanyang University, Seoul 04763, Republic of Korea; Department of Artificial Intelligence, Hanyang University, Seoul 04763, Republic of Korea; Department of Artificial Intelligence, Hanyang University, Seoul 04763, Republic of Korea; Department of Computer Science, Hanyang University, Seoul 04763, Republic of Korea

## Abstract

**Motivation:**

Accurately predicting protein function from sequence remains a fundamental yet challenging goal in computational biology. Although recent advances have enabled the reliable prediction of protein 3D structures from sequences, utilizing structural information alone for functional inference has shown limited success. To address this gap, previous work has explored the integration of sequence and structural data by representing proteins as graphs, where residues are modeled as nodes, and spatial proximity defines edges. However, since the number of amino acids can vary significantly between proteins, the resulting graphs, constructed based on amino acids, also differ greatly in size. This large variation poses a challenge, as it becomes extremely difficult to extract generalizable information from graphs of such differing scales accurately. In this work, we propose Structure-guided Sequence Representation Learning, a novel framework that incorporates structural knowledge to extract informative, multiscale features directly from protein sequences. By embedding structural information into a sequence-based learning paradigm, our method captures functionally meaningful representations more effectively. Furthermore, we present a generalizable model architecture designed for multitask learning and inference, offering improved performance and flexibility over traditional task-specific approaches to protein function prediction.

**Results:**

In this article, we demonstrate that the proposed novel attention pooling method on protein graphs effectively integrates global structural features and local chemical properties of amino acids in various-length proteins. Through this approach, we improve performance in tasks related to predicting protein functions, functional expression sites, and their relationships with structure and sequence. By effectively extracting the information needed to predict multiple protein functions simultaneously, we improve efficiency by eliminating the need for separate learning.

**Availability and implementation:**

The code implementation is available at https://github.com/vanha9/S2RL_protein and has also been archived on zenodo: https://doi.org/10.5281/zenodo.16441001.

## 1 Introduction

Accurate prediction of protein function is widely recognized as essential for understanding biological processes. Amino acid sequences provide the most fundamental and broadly accessible source of information, supporting functional inference through sequence similarity and the identification of conserved motifs or domains. However, even small variations in critical residues can lead to significant differences in molecular interactions or biological activity, meaning that high sequence similarity does not always guarantee functional equivalence. Moreover, sequence-based representations inherently lack spatial and interaction-specific information, which are critical for understanding protein functionality.

Structural information is also closely related to protein function and offers valuable spatial insight. Structural features—such as binding sites, spatial arrangements of residues, and domain-level folding patterns—provide key determinants for inferring protein function. However, structural similarity alone is also insufficient for accurate function inference, as minor changes in amino acid residues can still result in divergent behaviors. Moreover, the complexity and uncertainty associated with modern structure prediction algorithms like AlphaFold2 (Jumper *et al.* 2021) and RoseTTAFold (Baek *et al.* 2021) present practical limitations in directly using structural information for functional tasks. Therefore, integrating structural and sequence information is essential to overcome the limitations of using either modality alone.

**Figure 1.**
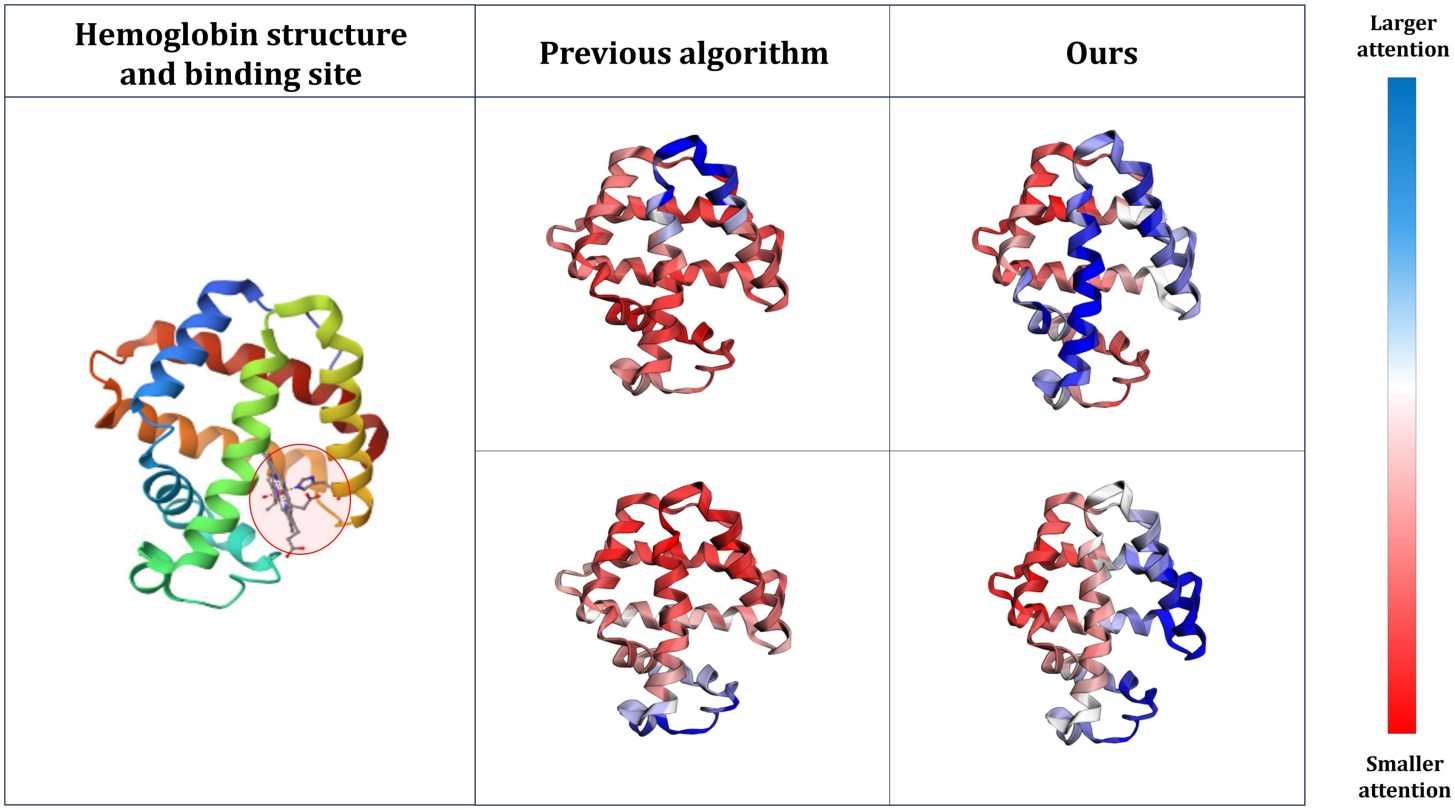
Attention weight visualization on hemoglobin structure. We compare the attention weights on the hemoglobin structure between the conventional cross-attention mechanism and our proposed algorithm. The conventional attention mechanism focuses on fixed-size regions, lacking variability in the scope of attention, and fails to attend to the heme group, which plays a crucial functional role. In contrast, our algorithm produces attention weights across multiple scales and assigns higher values to the actual heme-binding site, effectively capturing functionally important regions.

Previous studies, such as HEAL (Gu *et al.* 2023), have attempted to integrate sequence and structure information by modeling proteins as graphs, where nodes are defined from sequence information and edges are constructed based on structural proximity. These approaches typically employ graph convolution networks (GCN) (Kipf and Welling 2017) to capture structural relationships, often combined with cross-attention-based pooling techniques for function prediction. However, existing methods often fail to effectively capture meaningful features across multiple resolutions as shown in Fig. 1, particularly in proteins with diverse lengths and complexities. Moreover, GCN-based models are prone to over-smoothing, which can blur important amino-acid-level distinctions and reduce the effectiveness of these approaches in modeling critical structural and functional properties.

To address these challenges, we present Structure-guided Sequence Representation Learning (S2RL), a model that effectively extracts functionally relevant information from protein sequences, guided by structural knowledge. Our approach leverages 3D structure information to derive features across multiple structural resolutions—global, domain, and local levels—from sequences of varying lengths.

We construct residue-level graphs based on inter-residue distances and introduce a novel Structure-guided Sequence Representation Learning that leverages graph signal decomposition, derived through integration with the graph Laplacian. This approach explicitly integrates multiscale structural information and effectively extracts functionally meaningful representations from protein sequences of variable lengths. The design not only preserves local structural differences that are often lost in traditional GCN-based methods but also shows strong scalability to large and structurally diverse proteins. In addition, the model captures both structural and sequential characteristics of proteins without overfitting to specific tasks. As a result, it naturally supports multitask learning and achieves high performance as shown in Table 1. In general, we provide a unified framework for accurate and generalizable prediction of protein function.

## 2 Related works

### 2.1 Graph neural networks

With increasing interest in protein synthesis and drug discovery, AI-based approaches have been widely adopted for the prediction of protein functions, leading to the utilization of GNN-based approaches to effectively model 3D structural information. For example, DeepFRI (Gligorijevic *et al.* 2021) constructs a graph based on residue distances and applies GCN (Kipf and Welling 2017), while GAT-GO (Lai and Xu 2021) employs a GAT (Graph Attention Networks) (Veličković *et al.* 2018), incorporating self-attention among nodes. Following the successful debut of AlphaFold2 (Jumper *et al.* 2021), structural information—previously unavailable from sequence alone—can now be predicted with high accuracy. This advancement has facilitated the development of more complex and generalized models. Consequently, recent studies, such as Struct2GO (Jiao *et al.* 2023), have explored hierarchical encoding of GCN and self-attention, while HEAL (Gu *et al.* 2023) applies cross-attention between GCN-derived node features and task embeddings to enhance graph representation learning. Although GNN-based methods consider structural properties, they often fail to effectively capture globally distributed features and suffer from feature smoothing at the amino acid level due to local aggregation. This contradicts the biological significance of the amino-acid-level features, which play a crucial role in both protein structure and function.

### 2.2 Graph pooling

Graph pooling is a process that reduces the size of a graph, enabling efficient computation and making the model applicable to tasks such as graph classification, where the entire graph needs to be compressed into a single vector. Traditional pooling methods include mean pooling, which averages node features, and node selection-based pooling, which selects specific nodes to retain. Several approaches have been proposed to improve graph pooling. GPool (Gao and Ji 2019) learns the importance scores of the nodes and selects the top-*K* important nodes. SAGPool (Lee *et al.* 2019) extends this by incorporating GCN (Kipf and Welling 2017), preserving both node features and graph structural information. GAT-GO (Lai and Xu 2021) applies self-attention updates and sequentially prunes nodes based on attention weights. Struct2GO (Jiao *et al.* 2023) selects top-ranked nodes to form subgraphs and applies both sum pooling and max pooling. However, node selection-based approaches suffer from information loss, as even functionally important nodes may be discarded.

To address this, recent studies have focused on clustering-based pooling, where clusters are represented by super-nodes to aggregate information more effectively. DiffPool (Ying *et al.* 2018) introduces a soft assignment matrix, making the clustering process differentiable. HEAL (Gu *et al.* 2023) applies GCN-based graph updates and uses learnable queries as super-nodes, allowing each super-node to represent a distinct region of the graph for protein function prediction. Despite these advancements, these methods still struggle to fully capture multiscale features, which are essential for representing proteins due to the importance of both global structural relationships and local amino-acid-level interactions.

## 3 Materials and methods

In this section, we present an S2RL designed to capture multiscale intrinsic protein representations. This novel graph-based approach leverages structural information to extract functionally relevant features from sequences across multiple scales. The main idea of our algorithm is to represent the protein structure as a graph and decompose the connectivity among amino acids into a hierarchical structure consisting of global, domain, and local levels. After decomposing the graph based on different scales, we extract sequence embeddings corresponding to each scale and use them for function prediction. Furthermore, we introduce a multitask learning framework that enables the model to solve diverse functional prediction tasks through a single training process, without the need to train separate models for each task.

### 3.1 Preliminary

We begin by presenting the preliminary concepts and graph-related definitions necessary to understand the characteristics of our model.

Definition 1. *Simple graph is a pair* G=(V,E)*, where V is a set of elements called vertices and* E⊂{(x,y)|x,y∈V,x≠y}  *If* $(v_{1},v_{2})\in E$*, we call the element edge and* $v_{1}$  *is adjacent to* $v_{2}$.

Definition 2. *The degree* d(v)  *of a vertex v is the number of vertices in G that are adjacent to v, and the degree matrix D is a matrix with entry* $D_{i,j}\{\begin{array}{cc} d(v_{i}) & \text{if}\;i=j\\ 0 & \text{otherwise} \end{array}$.

Definition 3. *For given graph* G=(V,E)  *and*  $V=\{v_{1},v_{2},\ldots ,v_{n}\}$*, the Laplacian matrix* $L_{G}$  *of graph G is a* n×n  *matrix with each entry*  $L_{i,j}=\{\begin{array}{cc} -1 & \text{if}\;(v_{i},v_{j})\in E\\ d(v_{i}) & \text{if}\;i=j\\ 0 & \text{otherwise} \end{array}$

*and normalized Laplacian matrix of G is*  $D^{-\frac{1}{2}}LD^{-\frac{1}{2}}$  *where D is degree matrix of G.*

Given a graph G=(V,E) with *n* vertices and its corresponding normalized Laplacian matrix *L*, the smallest eigenvalue of *L* is 0, and its associated eigenvector is ${\left[1/\sqrt{n},1/\sqrt{n},\ldots ,1/\sqrt{n}\right]}^{⊤}$ where all components have the same value. Let the eigenvalues of *L* be denoted as $\lambda_{1},\lambda_{2},\ldots ,\lambda_{n}$, arranged in ascending order such that $0=\lambda_{1}\leq \lambda_{2}\leq \ldots \leq \lambda_{n}$ and denote their corresponding eigenvectors as $u_{1},u_{2},\ldots ,u_{n}$, respectively. Furthermore, as the eigenvalue increases, the variance of the components in the corresponding eigenvector also increases. This means that eigenvectors corresponding to larger eigenvalues capture more localized and high-frequency features, often resulting in significant differences between vertices. On the other hand, eigenvectors corresponding to smaller eigenvalues tend to assign similar values to vertices, regardless of their distance, encoding global features shared across vertices.

### 3.2 Model pipeline

**Figure 2.**
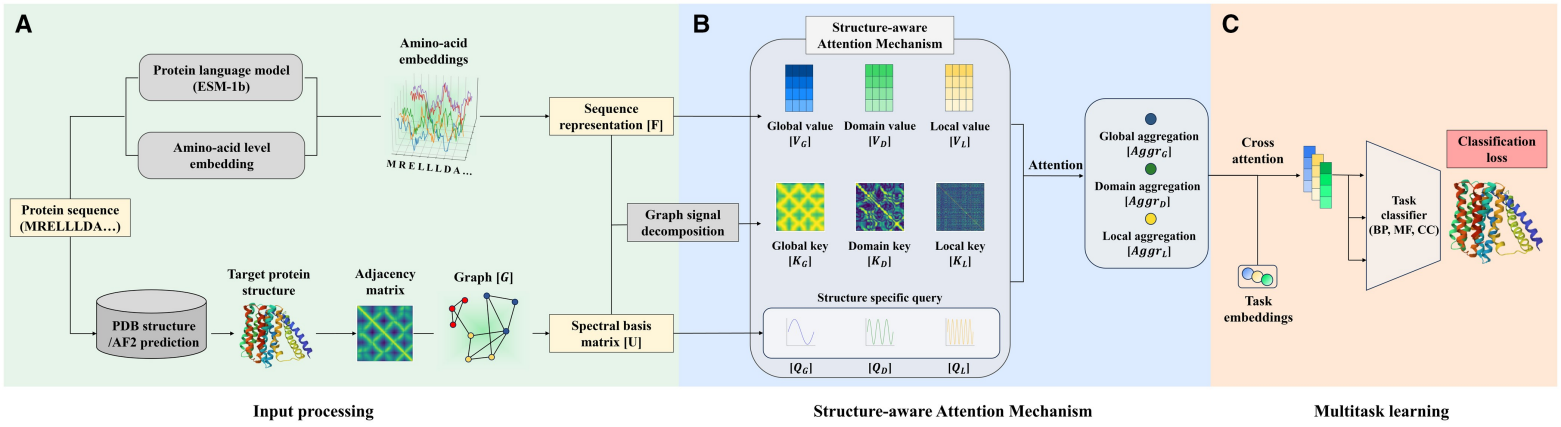
Overall pipeline of S2RL. (A) Input processing: To construct the model input, we utilize a pretrained protein language model to obtain amino acid embeddings. Additionally, we apply one-hot encoding to represent amino acid types, combining both representations to generate amino acid-level features. Each amino acid is represented by a *d*-dimensional feature vector, which can be viewed as *d* separate graph signals. These embedding vectors are stacked to form a matrix *F*, which represents the protein. Next, we construct a graph *G* by defining edges between amino acids that are spatially close based on their 3D structure. From this edge set, we compute the graph Laplacian matrix and extract its eigenvectors, which serve as a spectral basis to incorporate structural information. (B) Structure-aware Attention Mechanism: The proposed Structure-aware Attention Mechanism takes sequence representations and graph Laplacian eigenvectors as input and performs cross-attention. During key encoding, a graph signal decomposition is applied. As a result, the similarity between the encoded keys aligns with the distance map, as illustrated in the figure. This allows the attention mechanism to aggregate information at multiple structure levels. Additionally, we generate structure-specific queries by encoding Laplacian eigenvectors through a linear layer. This process ensures that the queries are tailored to the structural characteristics of each protein. Each query is designed to capture multiscale features, allowing the model to effectively capture global, domain, and local structural information. (C) Multitask learning: The processed graph representation serves as input to the model, passing through subsequent layers to perform multitask classification, capturing both structural and functional information.

In the previous section, we introduced the fundamental definitions related to graph structure and explored the spectral properties of the Laplacian eigenvalues and eigenvectors, which encode the structural information of a graph. In this section, we present the overall architecture and workflow of S2RL, focusing on how this idea can be applied to protein function prediction. The overall pipeline of our proposed model is illustrated in Fig. 2. Specifically, we describe a framework that utilizes both sequence and structure data to obtain embedding vectors and construct a structural graph. By leveraging the spectral characteristics of these graphs, we demonstrate how structural information relevant to function prediction can be effectively extracted from sequence-derived embeddings.

#### 3.2.1 Input data

As previously stated, the goal of this work is to enhance protein function prediction by jointly leveraging both sequence and structural information. Sequence information is represented using embedding vectors extracted from a pretrained protein language model, whereas structural information is incorporated through 3D atomic coordinates of the protein.

To obtain amino acid embeddings, we use ESM-1b (Rives *et al.* 2021), a protein language model trained with amino acids as tokens. Additionally, we generate node embeddings by combining these contextual embeddings with one-hot encoding of the 20 amino acid types. We incorporated one-hot encoding in our experiments to better reflect individual amino acid properties. One-hot encoding and ESM-1b embeddings are each projected to a common *d*-dimensional space via linear layers and then combined by summation. This additional channel helps preserve residue identity information that may otherwise be blurred by ESM contextualization, and, as shown in Table 2, improves robustness across different (p,q) thresholds. As a result, each amino acid is represented as a *d*-dimensional feature vector that integrates both sequence-derived contextual signals and residue identity information.

To incorporate structural information, we use experimentally determined structures from the Protein Data Bank (PDB) when available, and employ Alphafold-predicted structures for proteins without PDB entries. Based on these structures, we construct protein graphs by defining edges between amino acids whose pairwise distances fall below a predefined threshold.

After constructing the protein graph G=(V,E), consisting of *n* amino acids, we form a matrix $F\in R^{n\times d}$, where each row corresponds to the embedding vector of an amino acid, serving as the protein representation. We compute the graph Laplacian matrix *L* from the edge set *E*, which reflects the pairwise distances between amino acids. Let the eigenvalues of *L* be ordered as $0=\lambda_{1}\leq \lambda_{2}\leq \cdots \leq \lambda_{n}$, with corresponding eigenvectors $u_{1},u_{2},\ldots ,u_{n}$. We denote the matrix of eigenvectors as $U=\left[\begin{array}{cccc} u_{1} & u_{2} & \cdots & u_{n} \end{array}\right]\in R^{n\times n}$, which provides a spectral basis for analyzing the structure of the protein graph.

#### 3.2.2 Graph signal decomposition

We describe a method for extracting functionally relevant information by combining the matrix of embeddings *F* with the spectral basis *U* defined in the previous section. The core idea is to perform spectral analysis on the structural graph to identify multiscale structural patterns and retrieve the corresponding sequence signals from *F* at each scale.

By multiplying the two matrices $U^{⊤}$ and *F*, we can perform a transformation known as the Graph Fourier Transform, where the Laplacian eigenvectors serve as frequency components to transform the graph signal. This transformation allows us to represent the graph signal in the spectral domain. Each entry of the transformed graph signal is represented as follows.


(1) $${\hat{F}}_{i,j}=U_{i}^{⊤}f_{j}\;i\in \{1,2,\ldots ,n\},\;j\in \{1,2,\ldots ,d\}.$$

This means each component of the transformed signal represents the strength of the *i*th frequency component in the *j*th signal. In the context of proteins, this allows the representation of each amino acid to be decomposed according to structural frequency modes, where low-frequency components correspond to global or domain-level structural patterns and high-frequency components reflect localized variants such as residue-level environments.

By multiplying *U* with the transformed matrix, we can recover the original signal *F*.


(2) $$\begin{array}{c} F=U\hat{F}\\ =\left[\begin{array}{cccc} u_{1} & u_{2} & \ldots & u_{n} \end{array}\right]\left[\begin{array}{cccc} u_{1}^{⊤}f_{1} & u_{1}^{⊤}f_{2} & \ldots & u_{1}^{⊤}f_{d}\\ u_{2}^{⊤}f_{1} & u_{2}^{⊤}f_{2} & \ldots & u_{2}^{⊤}f_{d}\\ \vdots & \vdots & \ddots & \vdots \\ u_{n}^{⊤}f_{1} & u_{n}^{⊤}f_{2} & \ldots & u_{n}^{⊤}f_{d} \end{array}\right] \end{array}.$$

Each signal $f_{i}$, column of *F* can be interpreted and decomposed as follows:


(3) $$\begin{array}{l} f_{i}=\sum_{j=1}^{n}u_{j}(u_{j}^{⊤}f_{i}))\\ =\sum_{j=1}^{\left\lfloor n\times p\right\rfloor }u_{j}(u_{j}^{⊤}f_{i}))+\sum_{j=\left\lfloor n\times p\right\rfloor +1}^{\left\lfloor n\times q\right\rfloor }u_{j}(u_{j}^{⊤}f_{i}))\\ +\sum_{j=\left\lfloor n\times q\right\rfloor +1}^{n}u_{j}(u_{j}^{⊤}f_{i}))\;0<p<q<1 \end{array}.$$

**Figure 3.**
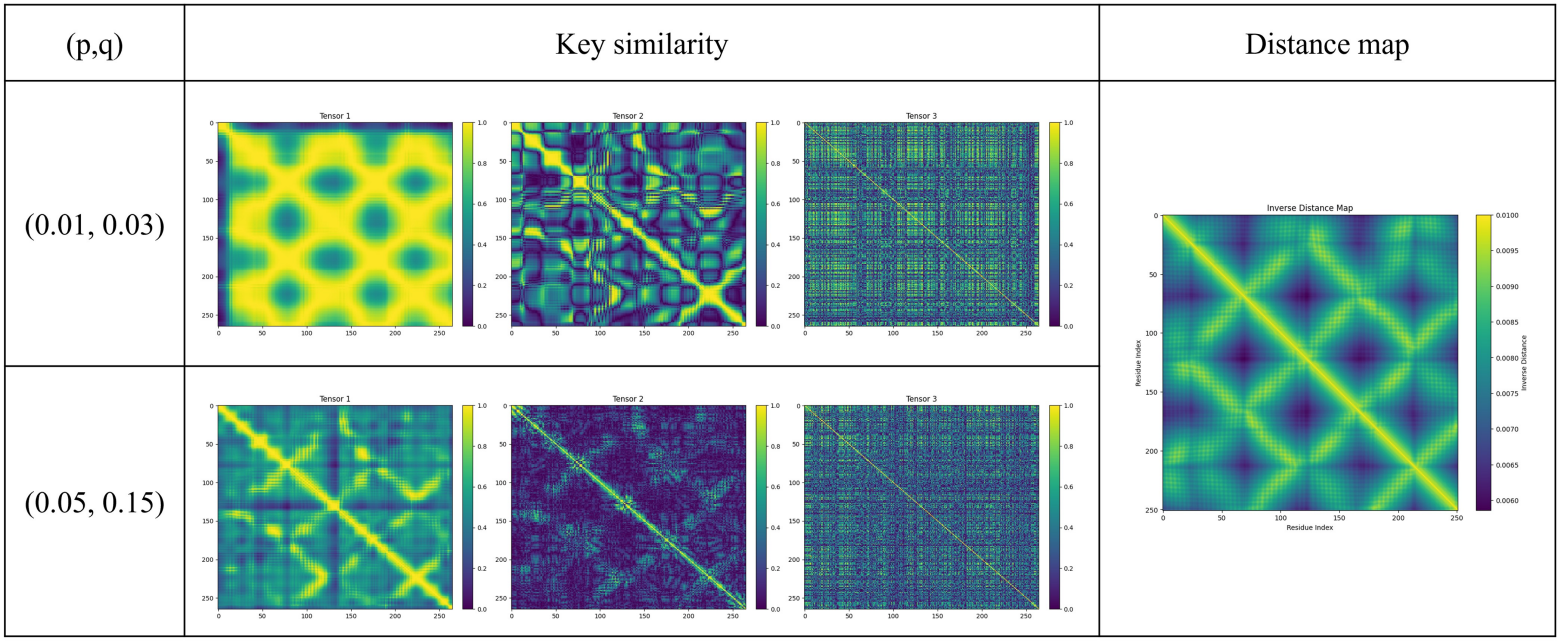
Effect of threshold parameters (p,q) on key similarity. Visualization of key similarity matrices ($K_{G}$, $K_{D}$, $K_{L}$) and the corresponding distance map under different (p,q) settings.

In our experiments, we tested multiple (p,q) pairs and fixed the values to p=0.01 and q=0.03 since larger (p,q) weakened the global scale aggregation as shown in Fig. 3. To faithfully leverage multiscale features, we therefore adopted (p,q)=(0.01,0.03) for evaluation.

Finally, the graph signal *F* can be decomposed as follows: three matrices


(4) $$\begin{array}{c} F={\left[\begin{array}{ccc} & | & \\ \cdots & \sum_{j=1}^{\left\lfloor n\times p\right\rfloor }u_{j}(u_{j}^{⊤}f_{i}) & \cdots \\ & | & \end{array}\right]}_{i\in \{1,2,\ldots ,d\}}\\ +{\left[\begin{array}{ccc} & | & \\ \cdots & \sum_{j=\left\lfloor n\times p\right\rfloor +1}^{\left\lfloor n\times q\right\rfloor }u_{j}(u_{j}^{⊤}f_{i}) & \cdots \\ & | & \end{array}\right]}_{i\in \{1,2,\ldots ,d\}}\\ +{\left[\begin{array}{ccc} & | & \\ \cdots & \sum_{j=\left\lfloor n\times q\right\rfloor +1}^{n}u_{j}(u_{j}^{⊤}f_{i}) & \cdots \\ & | & \end{array}\right]}_{i\in \{1,2,\ldots ,d\}}\\ =F_{G}+F_{D}+F_{L} \end{array}$$

named $F_{G},F_{D},F_{L}$, respectively.

Each column of the decomposed signal is represented as a weighted sum of the Laplacian eigenvectors, ordered by the size of their corresponding eigenvalues. According to the property of Laplacian eigenvectors, those associated with smaller eigenvalues tend to produce signals with similar values across columns. As a result, the row vectors are similar, representing globally smooth features. In contrast, signals composed of eigenvectors associated with larger eigenvalues display greater variance. While adjacent nodes may still share some similarity, the differences in signal values between distant or weakly connected nodes become more pronounced. Leveraging this property, we introduce an attention mechanism capable of capturing clusters at multiple scales, without relying on explicit clustering methods or GNN architectures. This approach effectively attends to different structural hierarchies in the graph representation of proteins.

#### 3.2.3 Structure-aware attention mechanism

Building on the previous section, we designed the Structure-aware Attention Mechanism. This module is designed to selectively aggregate information across different structural levels of protein by leveraging the spectral representation of the input protein graph. Specifically, we begin by selecting the Laplacian eigenvectors corresponding to the top *s* Laplacian eigenvalues, forming a spectral basis matrix $S\in R^{s\times n}$. To encode data-specific representations, we pass *S* through independent fully connected layers.


(5) $$Q_{G}=FC_{G}^{Q}(S),\;Q_{D}=FC_{D}^{Q}(S),\;Q_{L}=FC_{L}^{Q}(S),$$

where $Q_{G},Q_{D},Q_{L}\in R^{s\times c}$ denote the representation for the Global, Domain, and Local levels, respectively. Here, *c* is the dimensionality of the encoded representation space. These representations, called queries, serve as scale-specific descriptors that guide the attention mechanism in capturing relevant structural patterns at each level.

To generate keys that represent the structure-dependent features of each node at different scales, we perform graph signal decomposition on the node representations of the protein using Equation (4). The decomposed signals are then processed through individual fully connected layers to form the key K.


(6) $$K_{G}=FC_{G}^{K}(F_{G}),\;K_{D}=FC_{D}^{K}(F_{D}),\;K_{L}=FC_{L}^{K}(F_{L}).$$

For generating the value that carries the semantic content to be aggregated, we encode the protein graph signal *F* through separate fully connected layers dedicated to different scales, producing the value V.


(7) $$V_{G}=FC_{G}^{V}(F),\;V_{D}=FC_{D}^{V}(F),\;V_{L}=FC_{L}^{V}(F).$$

This design enables super-node pooling, where different super-nodes attend to distinct structural levels in the graph. Some super-nodes aggregate information from residues, capturing global structural patterns, while others concentrate on domain-level or local neighborhoods, informed by the underlying protein structure. This mechanism ensures that the resulting representations reflect both broad and fine-grained structural contexts. At each scale X∈{Global, Domain, Local}, the aggregation is performed using the attention mechanism defined as


(8) $$\begin{array}{c} \text{Attn}\_{\text{weight}}_{X}=\text{softmax}\left(\frac{Q_{X}\cdot K_{X}^{⊤}}{\sqrt{c}}\right)\\ {\text{ Aggr}}_{X}=\text{Attn}\_{\text{weight}}_{X}\cdot V_{X} \end{array},$$

where $Q_{X},K_{X}$, and $V_{X}$ are the query, key, and value representations associated with scale *X*, respectively. This aggregation serves as a summary representation of the amino acid features at the corresponding structural level, effectively capturing the hierarchical organization of the protein.

In summary, this process enables the aggregation of node representations in a graph that encodes a protein, where each node corresponds to an amino acid. In the case of global attention weights, the key vectors across all nodes are generated to be highly similar to one another. As a result, the attention weights that determine how information is combined are uniformly distributed across the entire graph. This leads to assigning nearly equal importance to all amino acids during aggregation, allowing the model to effectively capture globally distributed features that reflect the overall structural characteristics of the protein and general properties shared across amino acids. Conversely, for local attention weights, the keys exhibit high variance due to the decomposition process, and the property of the Laplacian eigenvector corresponds to a large eigenvalue. This variance causes attention weights to differ substantially between nodes, such that certain amino acids or local clusters receive much higher attention than others. This promotes more localized aggregation of information. Thus, global attention captures features distributed across the entire graph, providing a comprehensive representation of the overall structure. Local Attention selectively attends to specific amino acids and localized clusters, ensuring that region-specific and amino-acid-level features are effectively represented.

#### 3.2.4 Training

Based on the architecture, we obtain a feature representation denoted as ${\text{Aggr}}_{X}$ for each input protein. This representation is then fed into a classifier designed for protein function prediction, allowing for end-to-end training of the entire network based on a task-specific objective function. Specifically, we employ the standard cross-entropy loss for multi-label classification:


(9) $$L_{\mathrm{task}}=-\sum_{i=1}^{C}(y_{i}\;\text{log}\;{\hat{y}}_{i}+(1-y_{i})\;\text{log}(1-{\hat{y}}_{i})),$$

where *C* denotes the number of functional labels, $y_{i}\in \{0,1\}$ is the ground-truth label and ${\hat{y}}_{i}$ is the predicted probability for the *i*th class.

In our framework, the model is trained in a multitask learning setup that enables simultaneous prediction of multiple function types with a single training process. Since different functional categories, such as global, domain-level, or local annotations, may require different structural cues, our model extracts a task-dependent version of aggregation adapted to each functional objective. Training is thus performed by optimizing the task-specific objective in Equation (9) with respect to the aggregated features conditioned on the task.

To support task-specific feature aggregation, we incorporate a task embedding that represents the type of function prediction task and use it as an additional input to the aggregation mechanism.

## 4 Experimental results

For both training and testing, the same dataset and data splits as HEAL (Gu *et al.* 2023) are used for a fair comparison, which is available at https://github.com/ZhonghuiGu/HEAL.git. Following the conventional experimental settings, three multi-label classification problems are solved, which are biological process (BP), molecular function (MF), and cellular component (CC), to characterize the protein function from multiple perspectives. The MF ontology captures specific biochemical activities of proteins, such as catalysis and transport, at the molecular level. The CC ontology describes the subcellular locations where proteins operate, including structures like the cell membrane and mitochondria. The BP ontology contextualizes proteins within broader physiological processes such as DNA repair and signal transduction. Together, these ontologies—comprising 489 MF, 320 CC, and 1943 BP labels—provide a comprehensive framework for annotating protein function. To assess the performance of multi-label classification across these tasks, we employ three evaluation metrics: area under the precision-recall curve (AUPR), maximum F-score ($F_{\text{max}}$), and semantic distance ($S_{\text{min}}$).

We compared S2RL against both sequence-only methods, such as BLAST (Altschul *et al.* 1997), FunFams (Das *et al.* 2015), and DeepGO (Kulmanov *et al.* 2017), and structure-aware graph-based approaches, including DeepFRI (Gligorijevic *et al.* 2021), HEAL (Gu *et al.* 2023), and the recently proposed ensemble model TAWFN (Meng and Wang 2024). To further validate the impact of our design, we reproduced TAWFN under a multitask learning setting. As shown in Table 1, the experimental results demonstrate that models relying solely on sequence information exhibit limited performance, highlighting the importance of incorporating structural information for accurate function prediction. Compared to recent structure-aware graph-based methods, S2RL achieves competitive or superior performance across multiple tasks. Notably, as presented in Table 3, S2RL is trained using multitask learning, enabling efficient parameter sharing. As a result, S2RL achieves strong performance while maintaining the lowest parameter count among the recent approaches. Interestingly, the multitask learning variant of TAWFN has a larger number of parameters than S2RL, yet it performs worse in terms of predictive accuracy.

**Table 1.** Performance comparison between previous models and S2RL on the PDBch test set.^a^

Method	AUPR (↑)			Fmax (↑)			Smin (↓)			Sequence	Structure
	MF	BP	CC	MF	BP	CC	MF	BP	CC		
Blast [Altschul *et al.* 1997]	0.136	0.067	0.097	0.328	0.336	0.448	0.632	0.651	0.628	✓	✗
FunFams [Das *et al.* 2015]	0.367	0.260	0.288	0.572	0.500	0.627	0.531	0.579	0.503	✓	✗
DeepGO [Kulmanov *et al*. 2018]	0.391	0.182	0.263	0.577	0.493	0.594	0.472	0.577	0.550	✓	✗
DeepFRI [Gligorijevic *et al.* 2021]	0.495	0.261	0.274	0.625	0.540	0.613	0.437	0.543	0.527	✓	✓
HEAL [Gu *et al.* 2023]	0.688	0.334	0.446	0.735	0.610	0.700	0.340	0.506	0.459	✓	✓
TAWFN [Meng and Wang 2024]	**0.701**	**0.366**	0.471	0.726	0.605	0.700	**0.336**	0.499	0.454	✓	✓
TAWFN multitask	0.665	0.347	0.467	0.707	0.608	0.698	0.356	0.509	0.459	✓	✓
S2RL	0.676	0.350	**0.495**	**0.737**	**0.613**	**0.706**	0.353	**0.494**	**0.434**	✓	✓

a For each column, the highest performance is highlighted in bold.

**Table 2.** Evaluation of our proposed model under different threshold parameters (p,q) and with/without one-hot encoding.^a^

(*p, q*)	AUPR (↑)			Fmax (↑)			Smin (↓)			Use one-hot
	MF	BP	CC	MF	BP	CC	MF	BP	CC	
(0.01, 0.03)	0.676	0.350	0.495	0.737	0.613	0.706	0.353	0.494	0.434	✓
	**0.686**	**0.357**	**0.508**	**0.737**	**0.614**	**0.711**	**0.351**	**0.491**	**0.431**	✗
(0.025, 0.075)	**0.681**	**0.357**	**0.485**	0.735	**0.612**	0.693	**0.354**	**0.497**	0.446	✓
	0.672	0.346	0.472	**0.736**	0.610	**0.699**	0.356	0.498	**0.434**	✗
(0.05, 0.1)	0.671	**0.344**	**0.502**	**0.736**	**0.606**	**0.712**	**0.353**	0.498	**0.431**	✓
	**0.672**	0.343	0.484	0.728	0.605	0.703	0.356	**0.494**	0.436	✗

a The results demonstrate that incorporating one-hot encoding stabilizes residue identity and yields more robust performance, particularly when (p,q) increases. Bold indicates the best performance for each parameter.

**Table 3.** Comparison of the number of parameters across models.

Method^a^	The number of parameters			
	MF	BP	CC	Total
HEAL [Gu *et al.* 2023]	4.0M	5.4M	3.8M	13.2M
TAWFN [Meng and Wang 2024]	10.1M	12.4M	9.9M	32.4M
TAWFN multitask	–	–	–	15.5M
S2RL	–	–	–	**12.5M**

a Bold indicates the model with the fewest parameters.

**Figure 4.**
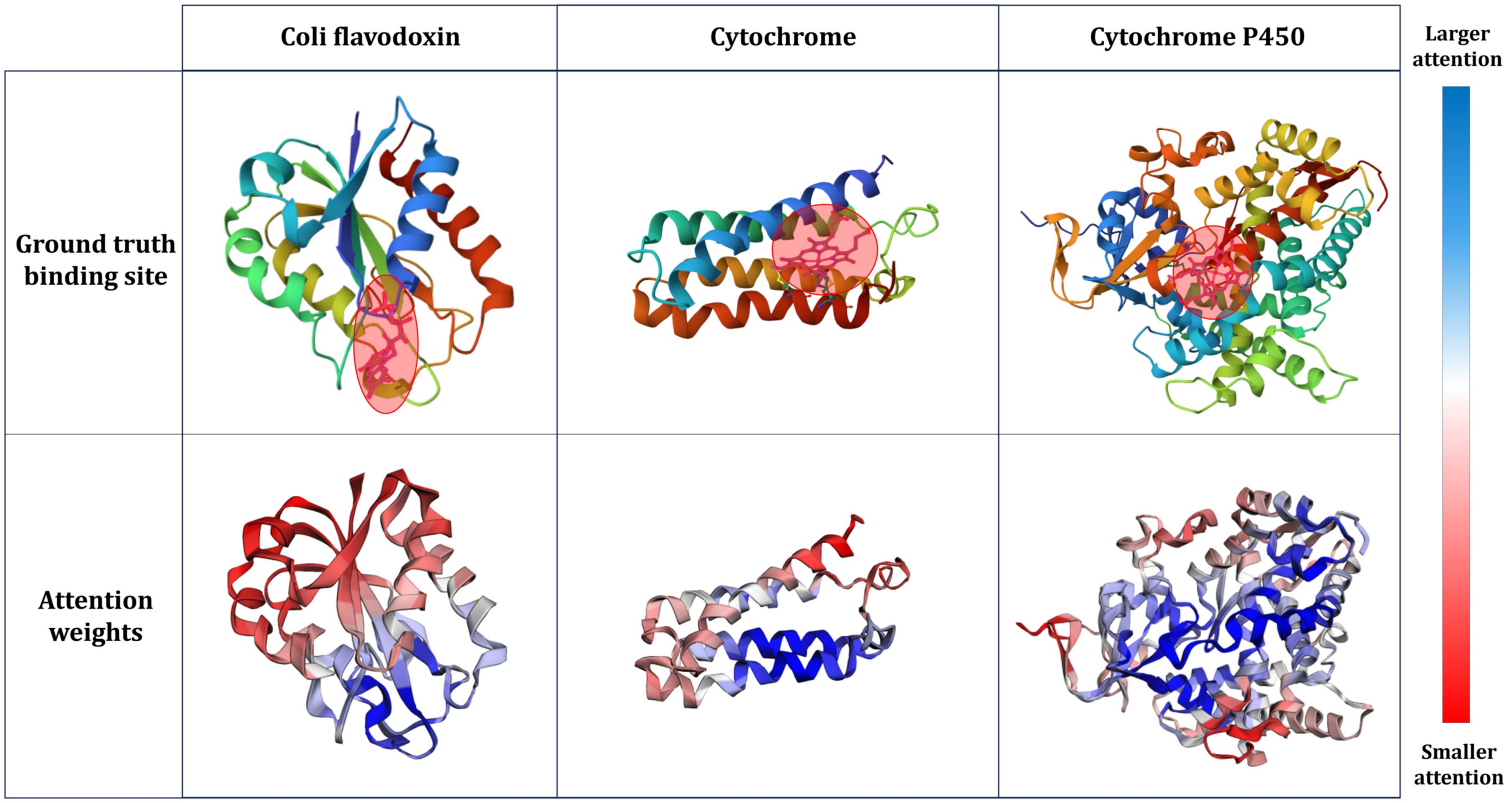
Visualization of the proposed model’s attention weights across proteins with varying lengths. High attention weights are observed around functional binding sites, reflecting the ability to encode structural context.

**Figure 5.**
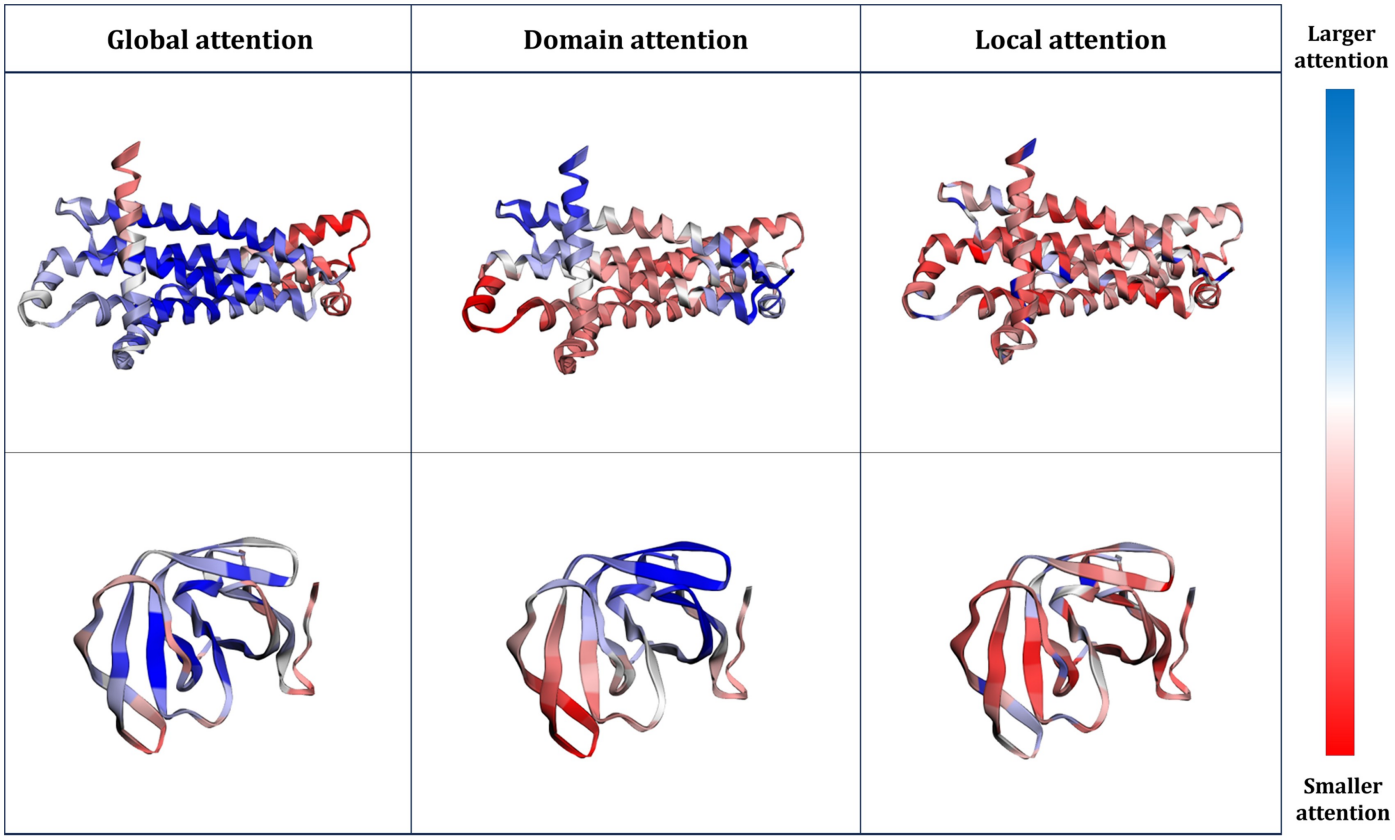
Visualization of the proposed model’s attention weights across multiple scales. Global attention captures global structural dependencies among amino acid groups, capturing overall structural relationships. Local attention focuses on individual amino acid residues, enabling fine-grained representation of functional sites. This demonstrates that our model aggregates information across multiple scales—from binding site clusters to individual amino acids—effectively integrating structural and functional context for accurate protein representation.

To validate whether our theoretically motivated design is effectively reflected in practice, we visualized the attention weights from multiple perspectives. In particular, Fig. 4. shows that attention scores are consistently higher at binding sites, suggesting that these regions receive more weight during the aggregation process and are therefore emphasized in the learned representation. Figure 5 illustrates the behavior of different scale attention mechanisms, revealing that global attention effectively combines long-range structure features, while local attention focuses on residue-level neighborhoods. These visualizations confirm that the model captures both global and local structural contexts necessary for accurate protein function prediction.

Our model is trained using multitask learning, where a single model learns to predict all tasks at once. On the other hand, the baseline models are trained separately for each task. Because of this difference, the comparison may not be completely fair. Even so, as shown in Tables 1 and 3, our model still shows competitive or better performance while using significantly fewer parameters than the baselines.

## 5 Conclusion

In this article, we propose a novel Structure-guided Sequence Representation Learning for protein function prediction, which effectively leverages hierarchical structural information to learn multiscale representations directly from protein sequences. Our approach allows for the aggregation of features from global structural clusters to individual amino acids, enabling the model to generalize across proteins of varying lengths and structural complexity. We address several key limitations of prior methods, including the lack of region-specific importance modeling, the inability to capture hierarchical context, and the over-smoothing of information across protein graphs. Our method preserves critical local signals while maintaining a global structural perspective.

Experimentally, we validate the capability of our model through diverse function prediction tasks without requiring task-specific retraining, highlighting its multitask generalization ability. Visualization of attention maps further confirms that our model attends to functionally relevant regions, demonstrating interpretable and biologically meaningful behavior.

Recent studies have proposed using diverse sources of information—such as protein structure, sequence, and textual descriptions—for protein function prediction. Methods such as FunBind (Boadu *et al.* 2025) and GOBeacon (Lin *et al.* 2025) tokenize protein structures (e.g., into 3Di tokens) to learn structural patterns. In contrast, our method preserves the sequence and leverages structure as a guide, providing graph connectivity and multiscale aggregation paths for the embeddings. These two directions are thus fundamentally different in their learning signals, but potentially complementary. For instance, 3Di tokens could be further refined with our structure-aware attention, or integrated as graph nodes alongside ESM and one-hot features to inject explicit structural-pattern information.

## Data Availability

Data is available at https://github.com/ZhonghuiGu/HEAL.git.
